# Next generation sequencing identifies a pathogenic mutation of *WFS1* gene in a Moroccan family with Wolfram syndrome: a case report

**DOI:** 10.1186/s13256-023-04150-2

**Published:** 2023-09-27

**Authors:** Maryem Sahli, Abdelali Zrhidri, Imad Boualaoui, Imane Cherkaoui Jaouad, Youssef El Kadiri, Yassine Nouini, Abdelaziz Sefiani

**Affiliations:** 1 Department of Medical Genetics, National Institute of Health in Rabat, BP 769 Agdal, 10 090 Rabat, Morocco; 2 Research Team in Genomics and Molecular Epidemiology of Genetic Diseases, Faculty of Medicine and Pharmacy, Genomic Center of Human Pathologies, Mohammed V University in Rabat, Rabat, Morocco; 3 Department of Urology A, Ibn Sina Hospital, Mohammed V University, Rabat, Morocco

**Keywords:** Wolfram syndrome, Next-generation sequencing, *WFS1* gene, Moroccan family

## Abstract

**Background:**

Wolfram syndrome is a rare autosomal recessive neurodegenerative disorder that affects 1/200,000 to 1/1,000,000 children. It is characterized by juvenile onset diabetes, optic nerve atrophy and other systemic manifestations. Symptoms of the disease arise mostly in early childhood with a high mortality rate due to severe neurological complications. Two causative genes have been identifed in this syndrome; the classical form is caused by autosomal recessive mutations of the *WFS1* gene, and a smaller portion of patients has mutations in the *CIDS2* gene, which are responsible for autosomal recessive Wolfram syndrome 2.

**Case presentation:**

We report the case of a 28-year-old Moroccan boy born from consanguineous parents referred to the department of medical genetics at the National Institute of Health in Rabat. The diagnosis of Wolfram syndrome was made based on insulin-dependent diabetes, optic nerve atrophy, sensorineural deafness, urological abnormalities and psychiatric illness. To establish the diagnosis at a molecular level, we performed next-generation sequencing in the index patient, which revealed compound heterozygous *WFS1* mutations: c.1113G > A (p.Trp371Ter) and c.1223_1224insGGAACCACCTGGAGCCCTATGCCCATTT (p.Phe408fs). This second variant has never been described in patients with Wolfram syndrome.

**Conclusion:**

The identification of the genetic substrate in our patient confirmed the clinical diagnosis of Wolfram syndrome and allowed us to provide him an appropriate management and genetic counseling to his family.

## Background

Wolfram syndrome (WS), is a rare autosomal recessive neurodegenerative disorder [1]. Its prevalence ranges between 1/68,000 and 1/770,000 [2]. It is characterized by various manifestations that can be summarized in the acronym DIDMOAD, including diabetes insipidus (DI), diabetes mellitus (DM), optic atrophy (OA) and deafness [2, 3]. Recently, some authors have extended the term DIDMOAD to DIDMOADUA due to the high association of urinary abnormalities (UA) [4]. Homozygous or compound heterozygous loss-of function mutations in *WFS1* gene on 4p16 are responsible for the majority of cases of WS [5]. Mutations in *CIDS2* gene on 4q24 have been described as an additional cause of WS in a few cases of Jordanian families [2, 6].

The prognosis of the WS is not favorable. Several patients have died in the third decade due to neurological complications or urinary dysfunctions, others because of the severe episodes of major depression that are accompanied by suicidal behaviors [4, 5, 7, 8]. A prompt diagnosis is important to provide proper early managements, to prevent or delay complications by routine ophthalmologic and urological screening tests [7].

Here we report the clinical and molecular analysis of a consanguineous Moroccan family with three WS affected children. Upon NGS, two non-sens variants were detected as compound heterozygous state in the WFS1 gene. One of the two mutations we found is novel. Our results confirm the clinical diagnosis of WS in this reported family and contribute to expand the mutational spectrum of this rare disease. Our study shows also, that next-generation sequencing (NGS) is a powerful and a cost-effective tool for the diagnosis of genetic disorder.

## Case presentation

### Patient

**Fig. 1:**
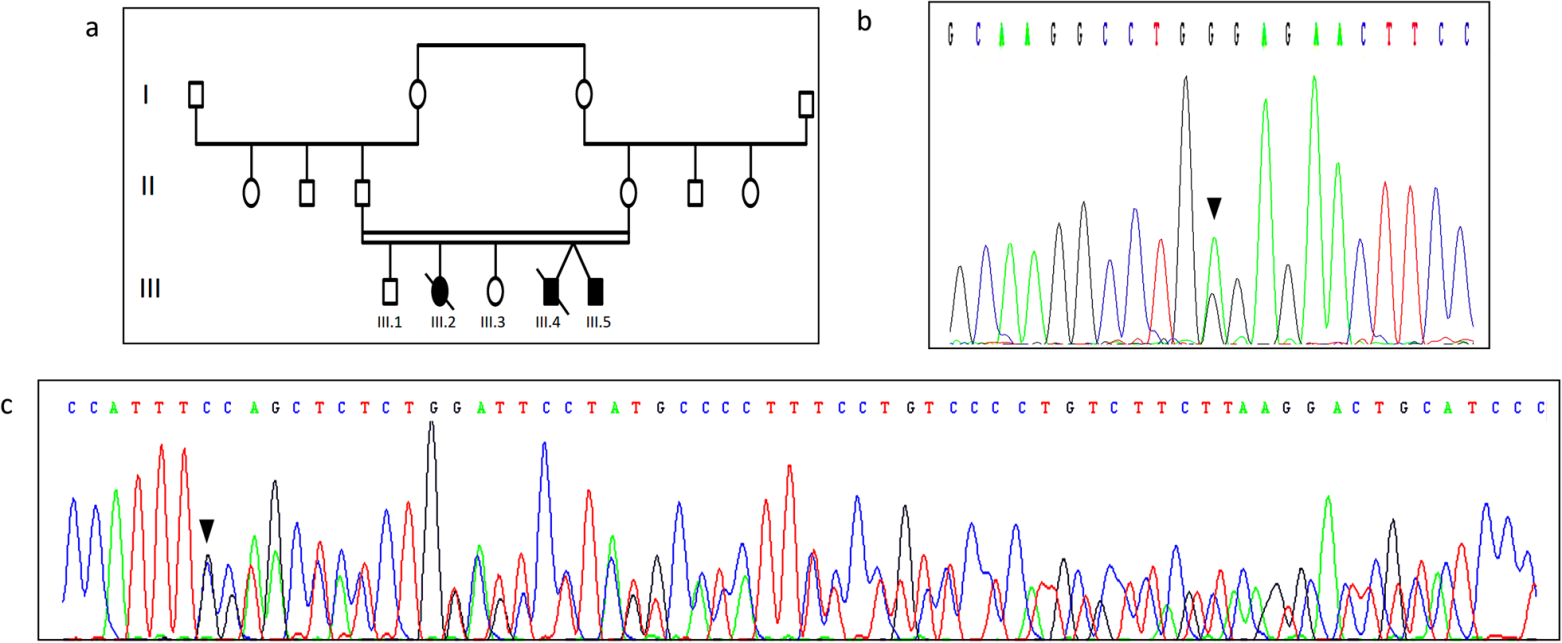
Pedigree of the Moroccan family with analysis of *WFS1* mutation. **a** Pedigree of the studied family. The filled symbol represents the affected patient and open symbols represent unaffected individuals. **b** Electropherogram showing the heterozygous mutation c.1113G > A (p.Trp371Ter) in the proband. **c** Electropherogram showing the heterozygous mutation c.1223_1224insGGAACCACCTGGAGCCCTATGCCCATTT (p.Phe408fs) in the proband. Black arrows indicate heterozygous mutation position detected in the proband

A 28-year-old Moroccan boy was referred by his urologist to our department of medical genetics at the National Institute of Health in Rabat for genetic diagnosis of Wolfram syndrome (Individual III.5; Fig. 1a). He was the last child of five siblings. The parents were first cousins and both are healthy. The patient was diagnosed at 7 years of life as having insulin-dependent diabetes mellitus and bilateral sensorineural deafness. Systematic eye examination showed optic atrophy.

During follow-up, growth hormone deficiency and recurrent urinary infections developed when he was 13 years old, a careful assessment for urinary tract showed bilateral hydronephrosis.

At the age of the genetic consultation, the patient presented neurological alterations with ataxia and cognitive impairment. The patient has also been diagnosed with severe anxiety and depression. His twin brother had a similar picture and died by suicide at the age of seventeen years (Individual III.4; Fig. 1a).

The patient’s 21-year-old sister; diagnosed type 1 diabetes mellitus and optic atrophy at the age of 10 years, died at the age of 21 years by a severe episode of hypoglycemia (Individual III.2; Fig. 1a).

### NGS and data analysis

The informed consent of patient was obtained prior to undergoing blood sample and carrying out molecular analysis. Genomic DNA was extracted from peripheral blood using commercial Invitrogen Thermo Fisher Scientific Kit (Pure LinkTM Genomic DNA Mini Kit-USA). DNA concentration and quality were determined. A customized panel was designed online using On Demand in Ion AmpliSeq Designer 6.1.3 (https://www.ampliseq.com/browse.action). We included in this panel 18 genes involved in different diseases from our consultation, among them the *WFS1* (NM_006005.3) gene responsible of Wolfram syndrome. The final customized panel was composed of average 532 amplicons divided into two primer pools and in silico covered 100% of regions of interest (ROI). This strategy is better adapted to our diversified requests of molecular tests, it remains a good strategy in public health system that facilitate the recruitment of candidates for the NGS by panel with a good cost-to-benefit ratio and reduced turnaround time and cost for multiple diagnostic tests. Libraries were prepared using Ion AmpliSeq Library Kit v2.0 (Thermo Fisher Scientific), according to the manufacturer’s instructions. One of 16 barcodes of the Ion Xpress Barcode Adapters1-16 Kit (Thermo Fisher Scientific) was added to each sample. Libraries were quantified with Qubit dsDNA HS Assay Kit on Qubit 2.0 Fluorometer (Molecular Probes). Equimolar amounts of each library were used to prepare template for clonal amplification and emulsion PCR was carried out on OneTouch2 Systems (Thermo Fisher Scientific) with Ion PGM HI-Q™ view OT2 Kit (Thermo Fisher Scientific). Templates were enriched using Ion OneTouch ES (Thermo Fisher Scientific) and prepared for 316v2 chip loading (Thermo Fisher Scientific). Finally, sequencing runs were performed on Ion Torrent Personal Genome Machine (PGM, Life Technologies) using Ion PGM HI-Q Sequencing, according to the manufacturer’s instructions.

Generated raw sequence data in FASTQ format were aligned to the hg19 human reference genome using the Torrent Mapping Alignment Program aligner implemented in v5.10.0 of the Torrent Suite software (Thermo Fisher Scientific). For SNV calling, we used plug-in Torrent Variant Caller v5.2.0.34 (Thermo Fisher Scientific) to generate a variant call format file. For Torrent Variant Caller analysis, default setting of germline low-stringency parameters (minimal variant frequency of 0.1, minimum variant quality of 10, minimum coverage of 5X, maximum strand bias of 0.98, and minimum variant score of 10) was used and candidate variants were obtained only when variant frequency at a given position of ≥ 20% and variant coverage of ≥ 20X.

Reported variants were confirmed in the Human Gene Mutation Database (HGMD, http://www.hgmd.cf.ac.uk/ac/index.php), Clinvar and previous publications. Amino acid predictions were performed using the SIFT algorithm, mutation Taster and PolyPhen2 software tools.

Upon NGS, we detected a compound heterozygous mutation c.1113G > A (p.Trp371Ter) and c.1223_1224insGGAACCACCTGGAGCCCTATGCCCATTT (p.Phe408fs) in exon 8 of the *WFS1* gene. Findings from NGS were confirmed by Sanger sequencing.

### Sanger sequencing

To confirm the mutation detected by NGS and to perform segregation analysis, Sanger sequencing was performed. Standard polymerase chain reaction (PCR) was carried on index case’s and parents’ DNA by using the forward 5′- AGG GTG GTC AGA GGG AGG -3′ and reverse 5′- GTA GGG CTC TGC ATG GGT G -3′ primer pair in the exon 8 of *WFS1* gene. PCR products were purified using ExoSAP and analyzed by standard Sanger dideoxy nucleotide sequencing using 3130 Genetic Analyzer (Thermo Fisher Scientific). Sanger sequencing confirmed the heterozygous mutation c.1113G > A (Fig. 1b) and the heterozygous mutation c.1223_1224insGGAACCACCTGGAGCCCTATGCCCATTT in the affected children (Fig. 1c).

## Discussion

Wolfram Syndrome (WS) is a rare genetic disorder initially described by Wolfram and Wagener in 1938 [9]. The exact prevalence of WS is not known, the birth incidence ranges from 1/68,000 and 1/770,000, depending on the population type and consanguineous populations [2, 10].

The current clinical manifestations of WS are mentioned in the acronym DIDMOAD which summarize the initials of the main clinical findings as follows: diabetes insipidus (DI), diabetes mellitus (DM), optic atrophy (OA) and deafness [2, 3]. In addition, there are other several systemic manifestations such as urinary abnormalities (hydroureter and hydronephrosis among others), neurological signs (ataxia, cognitive impairment), endocrine disorders (deficient growth hormone and corticotropin secretion, hypogonadism in man and delayed menarche in female), and psychiatric symptoms (ranging from mood swings, panic attacks and sleep abnormalities to severe depression) [7]. In fact, the association between DM and OA is the most common clinical finding of WS and the diagnosis should be suspected in the absence of either complete clinical findings [11].

WS is mainly caused by mutations in *WFS1* gene that are responsible for the classical form of Wolfram syndrome 1 (WS1) [5]. Mutations in *CIDS2* gene, which are responsible for Wolfram syndrome 2 (WS2) have been described as an additional cause in a few numbers of Jordanian families [2, 6]. WS1 and WS2 tend to have some different signs, diabetes insipidus and psychiatric disorders are absents in WS2, and gastrointestinal disorders (severe gastrointestinal ulcers) and hematological disturbances (bleeding and defective platelet aggregation) are only presents in WS2 [2, 6].

*WFS1* gene responsible for the most cases of Wolfram syndrome, was identified in 1998, maps to chromosome 4p16 and contains 8 exons (33.4 kb of genomic DNA) [12]. It encodes wolframin, a protein expressed in the endoplasmic reticulum and it has a role in regulation of intracellular calcium levels [3]. To date, approximately 427 distinct mutations have been characterized in patients with WS1 according to The Human Gene Mutation Database (HGMD) at the Institute of Medical Genetics in Cardiff (HGMD, http://www.hgmd.cf.ac.uk/ac/gene.php?gene=WFS1). Mutations are distributed throughout the gene with a hot spot in exon 8, by far the largest exon of *WFS1* gene containing 2.6 kb of DNA [13].

WS is inherited in an autosomal recessive fashion [1]. However, autosomal dominant mutations have been described to cause WS-like in families with less severe symptoms than those observed in Wolfram syndrome, with isolated adult-onset diabetes, isolated hearing loss, optic atrophy and hearing impairment, isolated congenital nuclear cataracts and no other manifestations of Wolfram disease [1, 14].

The genotype–phenotype correlation in WS patients has been intensively investigated; however, an univocal and clear consensus has not been yet reached. In fact, some researchers suggest that mutations creating a premature stop codon lead to the production of truncated proteins and consequently no functioning wolframin at the endoplasmic reticulum, and are correlated with a relatively severe clinical course with an early onset of diabete and optic atrophy. Moreover, mutations with residual protein expression in roughly associated with a milder disease course and consequently later onset of signs [3].

In this paper, we identified and characterized a previously unreported Moroccan patient with the clinical diagnosis of WS. The clinical features were consistent with those previously reported including diabetes mellitus, optic nerve atrophy, sensorineural deafness, urological abnormalities and psychiatric illness.

As a department of medical genetics providing genetic services and testing for various diseases in a country with lower income, molecular diagnosis of genetic diseases represents a real challenge. The recent introduction of NGS technology for the diagnosis in our lab bring us to consider customized multigenes panel approach of genes involved in the most common genetic diseases from our consultation and has allowed us to confirm the diagnosis of various genetic diseases. This strategy is better adapted to our diversified requests of molecular tests, it remains a good strategy in public health system that facilitate the recruitment of candidates for the NGS by panel with a good cost-to-benefit ratio and reduced the turnaround time and cost for multiple diagnostic tests.

Upon NGS, we detected compound heterozygous mutations c.1113G > A (p.Trp371Ter) andc.1223_1224insGGAACCACCTGGAGCCCTATGCCCATTT (p.Phe408fs) in exon 8 of the *WFS1* gene.

One of the genetic variants c.1113G > A (p.Trp371Ter) found in our patient has been identified previously in a homozygous state in several patients diagnosed with WS in the literature [4, 15]. While the second variant c.1223_1224insGGAACCACCTGGAGCCCTATGCCCATTT (p.Phe408fs) in *WFS1* gene was novel, unreported in population databases (ExAC, dbSNP, and 1,000 genomes), which was also predicted to be pathogenic by using Mutation Taster software. This second variant has not been previously described as a causative mutation in patients of WS to the best of our knowledge. The two mutations identified in our patient at heterozygous state are nonsense mutations leading to a truncated and non functional wolframin protein. Some studies have found that patients carrying compound mutation showed a variable phenotype. Patients carrying two mutations with the complete loss of function have been shown to have young onset of DM and OA than patients carrying two missense mutations with partial loss of function mutations [12, 16]. The clinical picture presented by our patient is broadly similar to that in other studies and fully exhibit the specific characteristics defined for WS patients carrying complete loss of function mutations.

Molecular diagnosis strategies by NGS have allowed confirming the diagnosis of WS, to provide an appropriate course of management to the patient, to identify and counsel heterozygous carriers predisposed to DM, sensorineural deafness and psychiatric illness. Furthermore, genetic diagnosis has allowed to offer adequate and timely genetic counseling, to discuss the available reproductive options, including prenatal diagnosis and preimplantation genetic diagnosis.

## Conclusion

We report here the clinical and molecular description of a Moroccan patient with WS. Upon NGS, two non-sens variants were detected as compound heterozygous state in the *WFS1* gene. One of the two mutations we found is novel; the results of our study will expand further the mutation spectrum of *WFS1* gene associated with WS.

## Data Availability

Available on request.

## Abbreviations

WS: Wolfram syndrome
NGS: Next-generation sequencing
DM: Diabetes mellitus
OA: Optic atrophy
WS1: Wolfram syndrome 1
WS2: Wolfram syndrome 2

## References

[1] Bansal V, Boehm BO, Darvasi A (2018). Identification of a missense variant in the WFS1 gene that causes a mild form of Wolfram syndrome and is associated with risk for type 2 diabetes in Ashkenazi Jewish individuals. Diabetologia.

[2] Duan L, Li Q, Tong A-L, Mao J-F, Yu M, Yuan T, Chai X-F, Gu F (2018). Clinical characteristics of wolfram syndrome in Chinese population and a novel frameshift mutation in WFS1. Front Endocrinol.

[3] Toppings NB, McMillan JM, Au PYB, Suchowersky O, Donovan LE (2018). Wolfram syndrome: a case report and review of clinical manifestations, genetics pathophysiology, and potential therapies. Case Rep Endocrinol.

[4] Bueno GE, Ruiz-Castañeda D, Martínez JR, Muñoz MR, Alascio PC (2018). Natural history and clinical characteristics of 50 patients with Wolfram syndrome. Endocrine.

[5] Li M, Liu J, Yi H, Xu L, Zhong X, Peng F (2018). A novel mutation of WFS1 gene in a Chinese patient with Wolfram syndrome: a case report. BMC Pediatr.

[6] Amr S, Heisey C, Zhang M, Xia X-J, Shows KH, Ajlouni K, Pandya A, Satin LS, El-Shanti H, Shiang R (2007). A homozygous mutation in a novel zinc-finger protein, ERIS, is responsible for Wolfram syndrome 2. Am J Hum Genet.

[7] Çelmeli G, Türkkahraman D, Çürek Y, Houghton J, Akçurin S, Bircan İ (2017). Clinical and molecular genetic analysis in three children with wolfram syndrome: a novel WFS1 mutation (c.2534T>A). J Clin Res Pediatr Endocrinol.

[8] Urano F (2016). Wolfram syndrome: diagnosis, management, and treatment. Curr Diab Rep.

[9] Sobhani M, Tabatabaiefar MA, Ghafouri-Fard S, Rajab A, Hojjat A, Kajbafzadeh A-M, Noori-Daloii MR (2020). Clinical and genetic analysis of two wolfram syndrome families with high occurrence of wolfram syndrome and diabetes type II: a case report. BMC Med Genet.

[10] Pallotta MT, Tascini G, Crispoldi R, Orabona C, Mondanelli G, Grohmann U, Esposito S (2019). Wolfram syndrome, a rare neurodegenerative disease: from pathogenesis to future treatment perspectives. J Transl Med.

[11] Maleki N, Bashardoust B, Zakeri A, Salehifar A, Tavosi Z (2015). Diabetes mellitus, diabetes insipidus, optic atrophy, and deafness: a case of Wolfram (DIDMOAD) syndrome. J Curr Ophthalmol.

[12] Rigoli L, Bramanti P, Di Bella C, De Luca F (2018). Genetic and clinical aspects of Wolfram syndrome 1, a severe neurodegenerative disease. Pediatr Res.

[13] Choi HJ, Lee JS, Yu S, Cha DH, Gee HY, Choi JY, Lee JD, Jung J (2017). Whole-exome sequencing identified a missense mutation in WFS1 causing low-frequency hearing loss: a case report. BMC Med Genet.

[14] De Franco E, Flanagan SE, Yagi T, Abreu D, Mahadevan J, Johnson MB, Jones G, Acosta F, Mulaudzi M, Lek N (2017). Dominant ER stress-Inducing *WFS1* mutations underlie a genetic syndrome of neonatal/infancy-onset diabetes, congenital sensorineural deafness, and congenital cataracts. Diabetes.

[15] Sobhani M, Amin Tabatabaiefar M, Ghafouri-Fard S, Rajab A, Mozafarpour S, Nasrniya S, Kajbafzadeh A-M, Noori-Daloii MR (2019). Clinical and molecular assessment of 13 Iranian families with Wolfram syndrome. Endocrine.

[16] Aloi C, Salina A, Pasquali L, Lugani F, Perri K, Russo C, Tallone R, Ghiggeri GM, Lorini R, d’Annunzio G (2012). Wolfram syndrome: new mutations, different phenotype. PLoS ONE.

